# Successful regression of metaplastic triple-negative breast cancer with neoadjuvant chemotherapy and surgical intervention: A case report

**DOI:** 10.1097/MD.0000000000042167

**Published:** 2025-04-18

**Authors:** Hadi Farhat, Razan Moghnieh, Yehya Tlaiss, Nour Bachaalany, Marwan Aoun, Elie Hage

**Affiliations:** a Department of General Surgery, University of Balamand, Faculty of Medicine and Medical Sciences, Dekwaneh, Beirut, Lebanon; b Department of Ophthalmology, University of Balamand, Faculty of Medicine and Medical Sciences, Dekwaneh, Beirut, Lebanon; c Department of Emergency Medicine, Hopital des Soeurs du Rosaire, Gemmayzeh, Beirut, Lebanon; d Department of General Surgery, Hopital des Soeurs du Rosaire, Gemmayzeh, Beirut, Lebanon.

**Keywords:** adjuvant chemotherapy, metaplastic breast carcinoma, neo-adjuvant therapy, radiotherapy, triple negative breast cancer

## Abstract

**Rationale:**

Metaplastic breast carcinoma (MBC) is a rare, aggressive subtype of triple-negative breast cancer, accounting for 0.2% to 5% of all breast cancers. It is associated with poor prognosis, twice the recurrence rate, and resistance to chemotherapy. This case report presents the treatment and outcome of a 39-year-old female with locally advanced stage III MBC.

**Patient concerns:**

The patient presented with a right breast mass, which had increased in size despite prior incomplete excision. She also had a contralateral breast mass, which was later confirmed as a benign neoplasm.

**Diagnoses:**

The patient was diagnosed with metaplastic carcinoma of the right breast, classified as triple-negative breast cancer due to the absence of estrogen, progesterone, and human epidermal growth factor receptor 2 receptors. A biopsy revealed mesenchymal differentiation with a Nottingham grade 3 tumor measuring 35 mm, with high mitotic index and positive surgical margins.

**Interventions:**

The patient underwent neoadjuvant chemotherapy with taxane/carboplatin, followed by modified radical mastectomy and axillary lymph node dissection on the right, and subcutaneous mastectomy with sentinel lymph node dissection on the left. Post-surgery, she received adjuvant chemotherapy and radiotherapy.

**Outcomes:**

Following neoadjuvant chemotherapy, significant morpho-metabolic tumor regression was observed. Postoperative imaging demonstrated near-complete resolution of the tumor and metastases. The patient’s quality of life improved significantly, despite the emotional and physical challenges of the treatment.

**Lessons:**

This case highlights the potential efficacy of combining neoadjuvant chemotherapy, surgery, and adjuvant therapy in the management of MBC, emphasizing the need for careful monitoring of clinical, histological, and imaging data to guide treatment decisions for this rare cancer subtype.

## 1. Introduction

Triple-negative breast cancer (TNBC) forms a diverse group of tumors displaying a range of histological compositions and prognoses.^[1]^ The rarest and most lethal of which is the metaplastic breast carcinomas (MBC), an infrequent and aggressive form of cancer consisting only 0.2–5% of all breast cancer cases.^[1]^ It is considered a TNBC because it lacks the expression of key receptors – estrogen receptor, progesterone receptor, and human epidermal growth factor 2 receptor.^[2]^ MBC carries the worst prognosis compared to other breast cancers playing a significant role in the overall global mortality attributed to breast cancer.^[2]^ MBCs are identified by the coexistence of a glandular epithelial element and a distinctive nonglandular metaplastic component, which include cell types exhibiting spindle, squamous, and/or sarcomatoid characteristics, such as chondroid or osseous attributes.^[1]^ Clinically, MBCs demonstrate increased metastatic potential and greater resistance to chemotherapy when compared with non-metaplastic TNBC, exhibiting twice the risk of recurrence and shorter periods of disease-free survival as well as overall survival.^[2]^ Due to the rarity and as a result of few clinical case reports present in the literature, there is a lack of standard treatment guidelines. However, downstaging with neoadjuvant therapy followed by surgical resection is the treatment of choice in TNBC.^[1]^ Additionally radical mastectomy is preferred over breast conserving surgery in MBC for several reasons including its inflammatory presentation, recurrence rate and overall survival.^[3]^ In this case report we present a case of a 39-year-old female patient, smoker, with bilateral breast masses, locally advanced stage III metaplastic TNBC on the right and another mass found to be a benign neoplasm on the left.

## 2. Case presentation

A 39-year-old female with a history of a right breast neoplasm presented for reevaluation due to concerns of recurrence after an incomplete excision with positive margins, as well as the presence of a contralateral breast neoplasm.

The patient’s history dates back to July 26, 2022, when she presented with a right breast mass. Her physician recommended a breast ultrasound, which revealed an irregular hypoechoic mass in the right breast, measuring 20 × 21 mm, located in the superoexternal quadrant at the 10 to 11 o’clock position, with no axillary lymphadenopathy. The left breast ultrasound showed 2 lateral periareolar hypoechoic nodules, measuring 13 × 9 mm and 14 × 11 mm, also without axillary lymphadenopathy. A biopsy was performed at the National Institute of Pathology, which revealed a nonconclusive histology with no malignant lesions at that time. The patient was discharged and scheduled for follow-up.

On September 26, 2022, the patient returned for a follow-up mammography, which revealed a well-defined opacity measuring 26 × 28 mm in the superoexternal quadrant of the right breast, indicating an increase in size. On October 18, 2022, she underwent a partial mastectomy of the right breast, and the histology was sent to the National Institute of Pathology. The histology results indicated metaplastic carcinoma with mesenchymal differentiation showing a heterogeneous Nottingham grade 3 component and a tumor size of 35 mm. The proliferation was composed of large polygonal cells showing marked cytonuclear atypia and prominent nuclei, arranged in trabeculae and lobules with occasional glandular structures. The stroma was fibrous, and the tumor cells were partially surrounded by a contracted matrix. The mitotic index was high, and there was the presence of perivascular lymphocytic exudate. The surgical margins were positive, and the tumor node metastasis (cancer staging system) classification (based on the 8th edition of American Joint Committee on Cancer) was T2.

In addition, the estrogen receptors, progesterone receptors and human epidermal growth factor receptor 2 were negative implying that her breast malignancy is triple negative. Subsequently, the patient was then put on a chemotherapy regimen of taxane/carboplatin.

On November 18, 2022, a positron emission tomography (PET) scan was performed, revealing postoperative changes consisting of a large focal area occupying both external quadrants of the right breast, extending near the outer edge of the right pectoralis major muscle with possible invasion. Additionally, hypermetabolic metastatic axillary lymph nodes (Level I) were detected on the right side. The updated tumor node metastasis (cancer staging system) staging was T4N++M0. One week later, a contrast-enhanced magnetic resonance imaging (MRI) was conducted. The patient continued her chemotherapy treatment.

On May 3, 2023, the patient underwent a follow-up MRI with contrast. Compared to the previous MRI, the results showed a partial but significant positive response. In the right breast, there was a decrease in the tumor process in the external quadrants and paralesional dermal infiltration, as well as a reduction in both micro and macronodular involvement in the upper quadrants and a decrease in axillary lymph node involvement. In the left breast, there was an almost complete reduction in regional micronodular involvement in the upper quadrants and a reduction in the size of 2 nodules (12 mm and 14 mm). Labs were ordered on May 6 and showed mild anemia with remarkable increase in the tumor marker cancer antigen 15-3 (Table 1).

**Table 1:** Patient labs before follow up PET scan.

Labs	Result	Unit	Reference values
CA 15-3	29.80	U/mL	0–26.4
Vitamin B12	614	pg/mL	187–946
TSH	0.743	uIU/mL	0.40–4.2
Red blood cells	3.65	10^6^/mm^3^	4.0–5.5
Hemoglobin	11.2	g/dL	12–16
Hematocrit	31.4	%	35–48
White blood cells	5000	mm^3^	3500-10,000
Neutrophils	63	%	40–65
Lymphocytes	23	%	20–40
Monocytes	11	%	2–8
Platelets	219,000	mm^3^	150,000-400,000
Creatinine	0.7	mg/dL	0.3–1.0
Total bilirubin	0.4	mg/dL	0.3–1.2
Direct bilirubin	0.0	mg/dL	0–0.2
SGOT	13	U/L	0–38
SGPT	13	U/L	0–45
Gamma-glutamyl transferase	29	U/L	0–55
Ferritin	76	ng/mL	10–180

CA 15-3 = cancer antigen 15-3 (tumor marker), PET = positron emission tomography.

The patient’s treatment plan involved 16 neoadjuvant chemotherapy sessions followed by surgery. The response to treatment was assessed through follow-up imaging studies. On May 8, 2023, a PET scan was performed, revealing significant improvements compared to the PET-CT scan from November 18, 2022. The large focal area in the right breast shows an almost complete morpho-metabolic response, with only a small, discreetly hypermetabolic area remaining at the junction of the external quadrants. The 2 other small lesions in the right breast have disappeared, and the previously hypermetabolic thickening of the skin has resolved. Metastatic axillary lymph nodes on the right side (Level 1) have nearly disappeared, with only 2 small hypermetabolic nodes persisting, while the nonspecific hypermetabolic nodes in the left axilla have disappeared. Regarding bone metabolism, there was no distant bone or visceral metastases.

After discussing the surgical treatment option with the patient on May 10, consent was given to opt for the surgery. The surgery was planned on May 19 and a modified radical mastectomy (MRM) with axillary lymph node dissection (ALND) on the right and subcutaneous mastectomy with sentinel lymph node dissection (SLND) on the left was performed. The operation was successful, and the patient underwent adjuvant chemotherapy with radiotherapy (RT). RT was opted for after 3 out of 9 right axillary lymph nodes turned out positive. Finally, left sentinel lymph nodes were negative.

The patient expressed satisfaction with the treatment outcome, noting a significant improvement in quality of life, although the process was emotionally and physically challenging. Written informed consent was obtained from the patient for the publication of this case report.

## 3. Discussion

The complexity of detection methods and the diverse range of diagnosis codes associated with these tumors pose a challenge in accurately assessing the incidence of MBC. Nevertheless, studies suggest that MBCs constitute between 0.2% to 1% of breast cancer cases in the United States, with variations depending on the specific classification employed.^[4]^ A significant majority of MBCs fall within the category of TNBCs, indicating the absence of progesterone, estrogen, and human epidermal growth factor receptor 2 receptors. In comparison to conventional triple-negative invasive tumors, MBCs exhibit notably bleaker prognoses, underscoring the urgency of targeted research and management strategies.^[2,5]^ MBC has twice as much of a chance of recurrence, and shorter disease-free and total survival times than non-metaplastic triple negative tumors.^[2]^

The prevailing histological subtype of MBC is squamous cell carcinoma, although it comprises a composite of ductal, squamous and/or chondroid, as well as spindle components. The World Health Organization (WHO) has further identified distinct subgroups within this spectrum, encompassing low grade adenosquamous carcinoma, fibromatosis-like metaplastic carcinoma, squamous cell carcinoma, spindle cell carcinoma, metaplastic carcinoma with mesenchymal differentiation, mixed metaplastic carcinoma, and myoepithelial carcinoma, thus delineating the intricate nature of MBC’s histopathology.^[5]^ The subgroup of the tumor in our case was metaplastic carcinoma with mesenchymal differentiation.

Histologically, MBC is characterized as an invasive carcinoma featuring both squamous and/or mesenchymal components, often highlighted by the presence of spindle cells and heterologous elements such as bone and cartilage. The proportion of squamous and mesenchymal elements can vary, with the potential for either component to dominate the tumor structure. Given this diverse composition, MBC frequently eludes accurate diagnosis or may be inadvertently disregarded during pathological assessments, mainly due to its inherent heterogeneity.^[6,7]^

In our presented case, the tumor exhibited distinctive characteristics, including large polygonal cells showcasing notable nuclear atypia, arranged in trabeculae and lobules, alongside occasional atypical glandular formations.

While MBC is recognized for its genetic heterogeneity and the presence of multiple somatic mutations, including TP53, PIK3CA, and PTEN, the absence of specific molecular markers capable of identifying MBC remains a challenge.^[5]^ Nevertheless, within the landscape of genetic variations, a notable enrichment of several components from the phosphatidylinositol 3-kinase/AKT/mammalian target of rapamycin (signaling pathway) pathway is observed in MBC compared to TNBC.^[8]^ This observation implies the potential viability of targeting this pathway as a therapeutic strategy for managing MBC. Furthermore, the presence of common mutations in beta-catenin, while subject to debate, has been sporadically identified through genomic profiling.

Frequent mutations and copy number alterations also extend to Receptor Tyrosine Kinases, including ERBB4, FLT3, and EGFR, indicating additional avenues for potential therapeutic intervention.^[8]^

Concerning the aspect of metastasis, a comprehensive study involving 136 patients diagnosed with MBC revealed distinctive patterns, with the lungs emerging as the most prevalent site of metastatic occurrences (n = 30, 22%). Following closely, metastases in the bone accounted for a significant portion (n = 18, 13%), while brain involvement was observed in a distinct subset (n = 9, 7%). Interestingly, the adrenal and spleen exhibited rarity in acting as metastatic sites, each registering a solitary instance within the patient cohort.^[9]^

Turning to treatment, neoadjuvant chemotherapy, administered prior to surgery, emerges as a key strategy. Nevertheless, the effectiveness of this approach has sparked debates within the medical community. In a notable study conducted by Yam et al., focusing on individuals afflicted by MBC, retrospective analyses of those subjected to neoadjuvant therapy unveiled rates of pathological complete response hovering around ~10%. This figure starkly contrasts with the more robust rates of 30% to 54% reported for non-metaplastic TNBC, prompting a discourse on the appropriateness of neoadjuvant chemotherapy for MBC patients. Indeed, this disparity has even led some voices to advocate against the adoption of neoadjuvant chemotherapy, and in certain cases, to reconsider chemotherapy as a standard course of action for individuals with MBC.^[8]^

Regarding surgical treatment, a comprehensive comparative study was conducted involving a cohort of 758 patients. Among these, 439 patients underwent breast conservative surgery combined with RT, while the remaining 319 patients underwent total mastectomy. The findings unveiled a notable contrast: the breast conservative surgery + RT treatment avenue demonstrated significantly enhanced overall survival rates among patients with MBC in comparison to the total mastectomy approach.^[10]^ However, in our specific case, the decision was made to MRM with ALND on the right and subcutaneous mastectomy with SLND on the left. This choice was driven by the grave prognosis, advanced staging, and presence of axillary metastasis, all of which collectively underscored the necessity for a more aggressive intervention strategy. The inclusion of RT proved to be of paramount importance in averting recurrence. Substantiating this, the effectiveness of RT has been conclusively demonstrated in enhancing overall survival post-surgery among patients afflicted with MBC.^[11]^

## 4. Conclusion

In summary, this case underscores the diverse challenges associated with managing MBCs, while also highlighting the remarkable response of MBCs to a comprehensive therapeutic approach involving neoadjuvant chemotherapy and surgery followed by adjuvant chemotherapy and radiotherapy. The patient’s unique circumstances and the aggressive nature of the tumor prompted a tailored strategy. This involved a MRM with ALND on the right side, complemented by a subcutaneous mastectomy with SLND on the left side. This decision was made in consideration of the ongoing debates surrounding various treatment regimens.

## Author contributions

**Conceptualization:** Hadi Farhat, Razan Moghnieh, Yehya Tlaiss, Nour Bachaalany, Marwan Aoun, Elie Hage.

**Data curation:** Hadi Farhat, Elie Hage.

**Formal analysis:** Hadi Farhat, Razan Moghnieh, Yehya Tlaiss, Marwan Aoun.

**Investigation:** Hadi Farhat, Razan Moghnieh, Yehya Tlaiss, Nour Bachaalany.

**Methodology:** Hadi Farhat, Elie Hage.

**Project administration:** Yehya Tlaiss, Marwan Aoun, Elie Hage.

**Supervision:** Marwan Aoun, Elie Hage.

**Validation:** Razan Moghnieh, Nour Bachaalany, Elie Hage.

**Visualization:** Yehya Tlaiss.

**Writing – review & editing:** Hadi Farhat, Razan Moghnieh, Nour Bachaalany.

**Writing – original draft:** Yehya Tlaiss.
